# K. ZHENG ET AL.Gold-nanoparticle-based multistage drug delivery system for antitumor therapy

**DOI:** 10.1080/10717544.2022.2128469

**Published:** 2022-10-13

**Authors:** Kaikai Zheng, Dong Zhou, Lili Wu, Jian Li, Bing Zhao, Shihao Zhang, Ruiying He, Lan Xiao, Iqbal Zoya, Li Yu, Yuhong Zhang, Yulin Li, Jie Gao, Kaichun Li

**Affiliations:** a Department of Oncology, Shanghai Fourth People’s Hospital, Tongji University School of Medicine, Shanghai, China; b Hubei Collaborative Innovation Center for Advanced Organic Chemical Materials, Key Laboratory for the Synthesis and Application of Organic Functional Molecules of Ministry of Education, Key Laboratory for the Green Preparation and Application of Functional Materials of Ministry of Education, College of Chemistry and Chemical Engineering, Hubei University, Wuhan, China; c School of Chemistry and Chemical Engineering, Nanchang University, Nanchang, China; d Changhai Clinical Research Unit, Shanghai Changhai Hospital, Naval Medical University, Shanghai, China; e Key Laboratory for Ultrafine Materials of Ministry of Education, Engineering Research Centre for Biomedical Materials of Ministry of Education, East China University of Science and Technology, Shanghai, China; f Institute of Health and Biomedical Innovation, Queensland University of Technology, Kelvin Grove Campus, Brisbane, Queensland, Australia; g The Australia-China Centre for Tissue Engineering and Regenerative Medicine (ACCTERM), Brisbane, Queensland, Australia; h Department of Trauma Orthopedics and Microsurgery, Zhongnan Hospital of Wuhan University, Wuhan, China

**Keywords:** Alginate, gold nanoparticle, pH/redox/enzyme responsiveness, nanogel, multistage drug delivery, antitumor therapy

## Abstract

Nanoparticles can promote the accumulation of drugs in tumors. However, they find limited clinical applications because they cannot easily penetrate the stroma of cancer tissues, and it is difficult to control drug release. We developed a multiresponse multistage drug-delivery nanogel with improved tumor permeability and responsiveness to the tumor microenvironment for the controlled delivery of anticancer agents. For this purpose, ∼100 nm multistage drug delivery nanogels with pH, redox, near-infrared stimulation, and enzyme responsiveness were grown in situ using 20 nm gold nanoparticles (AuNPs) via an emulsion-aiding crosslinking technique with cysteine crosslinker. An alginate cysteine AuNP (ACA) nanocarrier can efficiently load the cationic drug doxorubicin (DOX) to produce a multistage drug delivery nanocarrier (DOX@ACA). DOX@ACA can maintain the slow release of DOX and reduce its toxicity. In cancer tissues, the high pH and reductase microenvironment combined with the in vitro delivery of alginate and near-infrared light drove drug release. The developed nanoparticles effectively inhibited cancer cells, and in vivo evaluations showed that they effectively enhanced antitumor activity while having negligible in vivo toxicity to major organs.

## 1. Introduction

The systemic administration of nanoparticles into tumors is usually governed by the enhanced permeability and retention (EPR) effect (Liu et al., 2019; Ding et al., 2020; Tu et al., 2020). Most currently available nanoparticles cannot sufficiently penetrate cancer cells to release drugs into them, and thus, they find limited clinical applications (Wang et al., 2020). Nanoparticle drug delivery usually involves three steps: nanoparticles reach the tumor site via vascular transport, pass through the tumor blood vessel wall, and penetrate the stroma to release drugs into the cancer cells (Ruan et al., 2015; Liu et al., 2018). Tumor blood vessels usually show high density around the tumor, low density at the central position, and an abnormal pore wall (range: 10–2000 nm) (Vaahtomeri & Alitalo, 2020; Sheth et al., 2021). Furthermore, abnormally proliferating cancer tissues compress blood and lymphatic vessels, leading to increased fluid pressure (Curley et al., 2020; Shu et al., 2021). Finally, owing to the high permeability of the tumor vascular system, collagen is abnormally expressed in cancer tissues (Han et al., 2020). In particular, the tumor collagen matrix has a higher density than normal tissue (Liu et al., 2020). The above factors, namely, heterogeneous structures, increased fluid pressure, and compact extracellular matrix of solid tumors, prevent the spread of nanoparticles in them (Haase et al., 2020; Skotland & Sandvig, 2021).

Tumor penetration can be enhanced by controlling the properties of the nanoparticles (e.g. size, configuration) (Ryu et al., 2020; Hu et al., 2021). Among the various properties, size is the key one. The permeability efficiency of nanoparticles is negatively correlated with their size (Jin et al., 2019). Specifically, smaller nanoparticles show higher tumor penetration (He et al., 2020). However, small nanoparticles have a short half-life and are quickly cleared by the kidney; furthermore, they may penetrate normal tissues and cause adverse reactions (Han et al., 2021). Therefore, the initial nanoparticle size should be relatively large to allow for a long circulation time; however, once the nanoparticles enter the stroma of the cancer cell, their permeability should be increased (Yu et al., 2019). To satisfy these conflicting requirements, stretchable nanoparticles that can respond to different tumor microenvironment stimuli have been developed. Such nanoparticles have a long circulation time and can thus accumulate through EPR in the blood. Then, near the tumor area, they release smaller nanoparticles (<50 nm) that can easily pass through the tumor parenchyma and reach all cancer cells in sufficient concentrations for effective therapy (Yu et al., 2020; Lv et al., 2021).

Drug release via tumor-site-specific microenvironmental stimulation of nanoparticles is also considered an effective treatment (Chen et al., 2020; Jia et al., 2021). However, the typical stimulation is insufficient and uneven; therefore, drug release cannot be controlled effectively (Anderson & Simon, 2020; Boedtkjer & Pedersen, 2020). In recent years, external near-infrared (NIR) stimulation for noninvasively activating nanoparticles to release drugs and to produce a high temperature has emerged as an effective treatment (Ou et al., 2019). Resonance light scattering in gold nanoparticles (AuNPs) produces a good photothermal effect, and AuNPs can respond to external light stimuli, promote drug release, and produce heat; therefore, they have been applied for the photothermal therapy of cancer (Volchan et al., 2017; Wang et al., 2020).

**Scheme 1.**
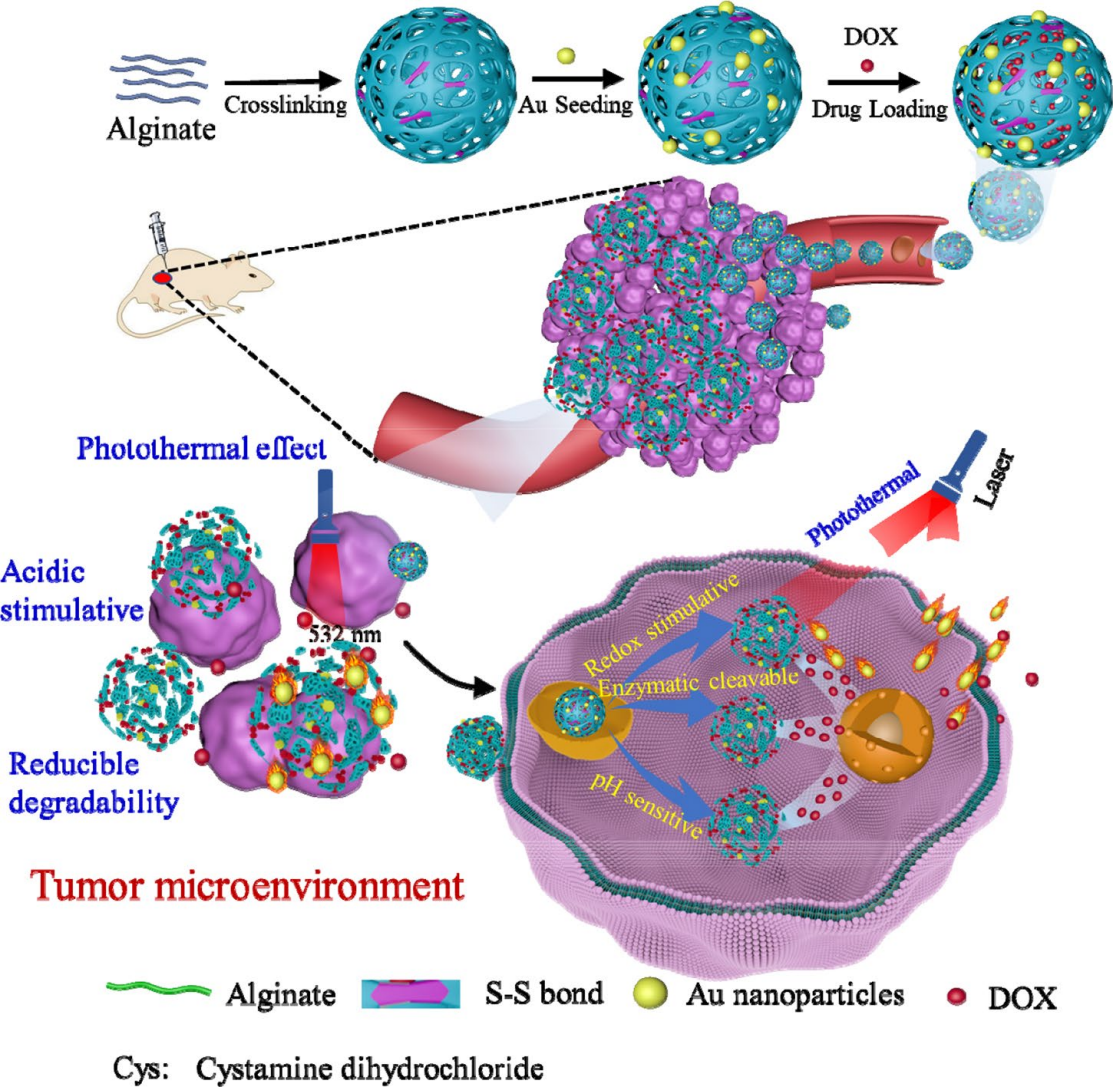
Schematic diagram of the development of nanogels as a novel selective drug delivery method for antitumor therapy.

Alginate (Alg) is a bioactive natural polysaccharide with good biocompatibility and degradability, pH and alginate lyase responsiveness, and immunostimulatory properties; therefore, it shows promise as a nanocarrier (Fay et al., 2010; Jahanban-Esfahlan et al., 2020). However, alginate lacks sufficient stability. Alginate can form a solid three-dimensional structure through crosslinkers to maintain stable drug delivery (Zhou et al., 2018; Chiu et al., 2020). Herein, we aimed to design a controllable drug delivery system that enhances tumor penetration and cellular uptake while maintaining stable in vivo circulation through the tumor-mediated release of small nanobubbles; endogenous tumor-specific signals and exogenous signals enhance intracellular drug accumulation through delivery (Zhou et al., 2020). Specifically, we developed multiple-response drug-delivery nanogels based on our previously developed multiple-response nanogels and the photothermal ability of AuNPs. Alginate and cystamine dihydrochloride, a chemical crosslinking agent with redox degradation ability, were chemically crosslinked and polymerized to form nanogels by the double emulsion method. AuNPs were then loaded in situ onto the nanogel to realize increased therapeutic efficacy. Nanogels can degrade and release drugs in the tumor microenvironment through pH and redox responses. Drug release can also be controlled with high efficiency through NIR irradiation heating and sodium alginate lyase. In vivo experiments showed that the developed nanogels afforded increased therapeutic efficacy compared with free drugs. Mice treated with these nanogels showed higher tumor inhibition rates and a longer lifespan. These nanogels have low toxicity and high therapeutic efficacy, making them promising for clinical applications (Scheme 1).

## 2. Materials and methods

### 2.1. Materials

1-(3-Dimethylaminopropyl)-3-ethylcarbodiimide hydrochloride (EDC), D, L-dithiothreitol (DTT), and polyvinyl alcohol (PVA, Mw: 72,000 Da) were obtained from Sinopharm Chemical Reagent Co., Ltd. (Shanghai, China). Doxorubicin (DOX), a cationic anticancer drug, was purchased from Dalian Meilun Biotechnology Co., Ltd. (Dalian, China). Alginate lyase was purchased from Hubei Xinmingtai Chemical Co., Ltd. (Wuhan, China). Alginate acid sodium salt (from brown algae, Mw: 12–58 kD, cell culture tested), dioctylsulfosuccinate (AOT), cystamine dihydrochloride (Cys), and gold(III) chloridehydrate (HAuCl_4_·3H_2_O) were purchased from Sigma–Aldrich (USA).

### 2.2. Preparation of Alg/Cys nanogels

Alginate nanogels were prepared by the double emulsification method (Rawat & Saraf, 2009). Alginate solution (2 g, 1–2 wt%) was added to the EDC solution (1 mL, 5.5 mg/mL) and then stirred for 3 h at 30 °C. This solution was then added to the AOT solution (4 mL, 2.5 wt%) in dichloromethane with stirring. The resulting solution was stirred for 5–10 min. Then, 2 wt% aqueous PVA solution (weight: 60 g) was added with stirring at 400 rpm for 10 min. Next, Cys (0.1 mg/mL, 4 mL) was dropped into the mixed solution and stirred overnight to evaporate dichloromethane from the solution. The resulting solution was centrifuged at 12,000 rpm for 15 min and washed with water 2–3 times to obtain a viscous precipitate that was freeze-dried for 24 h to obtain Alg/Cys nanogels.

### 2.3. Preparation of Alg-AuNP nanogels

A 0.01 M aqueous solution of HAuCl_4_ was added to 1 mL of the nanogels. The mixture was ultrasonicated (JM-07) for 20 min as a result of which it quickly turned into a yellowish dispersion. Cetyltrimethylammonium bromide (CTAB, 0.1 M) was slowly added to the dispersion, and ice-cold NaBH_4_ was subsequently added dropwise to the dispersion. To prepare the growth solution, 0.01 M HAuCl_4_ solution was added to the prepared 0.1 M CTAB (5 mL), which was sonicated at room temperature for 10 min and then added to 0.01 M AgNO_3_ solution. Finally, 0.1 M ascorbic acid (AA) was slowly added with stirring, following which it was left to stand overnight.

### 2.4. Drug loading and release study

DOX solution (1 mL, 2.0 mg/mL) was mixed with 5 mL of the nanogel solution (50.0 mg/mL). After stirring for 12 h, the remaining DOX was removed with a dialysis membrane (molecular weight cutoff: 8000–14,000 DA) to obtain DOX@ACA nanogels. The absorbance of the dialysate was measured using an ultraviolet spectrophotometer (UV-61000S) at 490 nm, and the DOX content was calculated. Then, 1 mL of DOX@ACA carrier solution was added to different simulation solutions: pH (5.0, 6.5, and 7.4), with or without DTT (5.0 and 10.0 mM), and/or alginate lyase (0.5 and 1.0 mg/mL). The drug release under 532 nm laser irradiation was studied. The drug release at different time points was determined using ultraviolet spectrophotometry. The drug release of the nanogels was calculated as

(1) $$Cr\left(\%\right)=Ab_{st}/Ab_{\mathrm{stot}}\times 100\%$$

where *Ab_st_* is the total amount of drug released at time *t*, and *Ab_stot_* is the total amount of drug initially contained in the nanogel.

### 2.5. Nanogel characterization

The functional groups of the nanogels were characterized by Fourier transform infrared spectroscopy (Perkin-Elmer, USA) and Raman spectroscopy (Renishaw, France). The morphology of the nanogels was evaluated by transmission electron microscopy (TEM, Tecnai G20, USA). Before testing, a sample was dropped onto the copper wire of the carbon supporting film and placed in a fume hood for air-drying overnight. The hydrodynamic diameter and surface charge of the nanogels in phosphate-buffered saline (PBS) were measured using a Zetasizer (Nano ZS, Malvern Instruments).

### 2.6. In vitro biocompatibility and toxicity

A549 human lung adenocarcinoma cells were treated with ACA nanogel. Then, a cell count kit 8 (CCK-8) was used to evaluate the cell biocompatibility and toxicity. A549 cells were treated with fresh Dulbecco’s modified Eagle medium (DMEM) containing free DOX (concentration: 0.5, 1.0, 2.0, 2.5, and 3.0 μM), ACA, and DOX@ACA. Cells treated with ACA and DMEM without DOX were used as controls. After 48 h, the cell biocompatibility and toxicity were measured using CCK-8. The optical density (OD) of the 96-well plate was measured at 450 nm. The cell viability was calculated as

(2) $$\mathrm{Cell}\mathrm{Viability}\left(\%\right)=OD_{eg}/OD_{cg}\times 100\%$$

where *OD_eg_* and *OD_cg_* are the OD of the number of viable cells in the experimental and control groups, respectively.

### 2.7. In vivo antitumor evaluation

C57BL/6J mice (female, ∼20 g, 6 weeks old) were purchased from Liaoning Changsheng Biotechnology Co., Ltd. (Liaoning, China) for the study. The experimental protocols were approved by the Institutional Animal Care and Use Committee of the Animal Experiment Center of Wuhan University of Chinese Medicine (Wuhan, China). were bred in a suitable environment and divided into three groups of five each (tumor size: 200 mm^3^). The mice were treated by the intraperitoneal injection of PBS, DOX@ACA, and DOX once every two days. The final DOX content was 4 mg/kg per mouse, and the weight of the mice and volume changes of the tumor were recorded. The tumor volume was calculated as

(3) $$V=\left(\left(\mathrm{tumor}\mathrm{length}\right)\times {\left(\mathrm{tumor}\mathrm{width}\right)}^{2}\right)/2$$

where *V/V_0_* is the relative tumor volume ratio; *M/M_0_* is the relative body weight ratio; *V_0_* is the tumor volume of mice before injection; and *M_0_* is the tumor weight of mice before injection.

### 2.8. In vivo biocompatibility

Tumor, liver, heart, spleen, and kidney sections were collected for histological observation with hematoxylin and eosin (H&E) staining. The tissues were fixed, dehydrated, embedded in paraffin, and cut into 6 μm slices using a tissue slicer (Leica RM2265, Germany). The tissue sections were stained with H&E and then imaged using a fluorescence inverted microscope (Leica DMI8, Germany).

### 2.9. Tumor cell apoptosis evaluation

Tumor cell apoptosis was detected using a terminal deoxynucleotidyl transferase-mediated deoxynucleotide triphosphate nick-end labeling (TUNEL) bright green apoptosis detection kit (G3250, Promega Biotech Co., Ltd., USA) according to the manufacturer’s instructions. Nuclei were detected using a DS-U3 fluorescence microscope (Nikon, Japan). The samples were observed using a microscope at 400x magnification.

## 3. Results and discussion

To overcome drawbacks such as the lack of response or inaccurate drug delivery by endogenous stimuli in single-response nanoplatforms, we developed multiple-response composite nanogels to achieve high therapeutic efficacy and efficiency. Alginate, which showed pH and enzyme response, was selected as the matrix for composite nanogels (Bauleth-Ramos et al., 2019; Mirrahimi et al., 2019). Alginate was crosslinked with cysteine, thereby additionally endowing the nanocarriers with a redox response (Zhang et al., 2017) and improving the colloidal stability of the nanogels. To further enhance the therapeutic efficacy, AuNPs with excellent photothermal effects were attached to the nanogels. DOX was loaded onto the multiple-response nanogels (DOX@ACA). DOX@ACA nanogel fabrication was driven by hydrogen bond formation among DOX, alginate, and AuNPs. AuNPs were attached to the nanogels through electrostatic interactions. Additionally, the electrostatic interaction between DOX and alginate enabled the former to be effectively encapsulated into the nanogels.

### 3.1. Structural characterization of ACA nanogels

**Figure 1.**
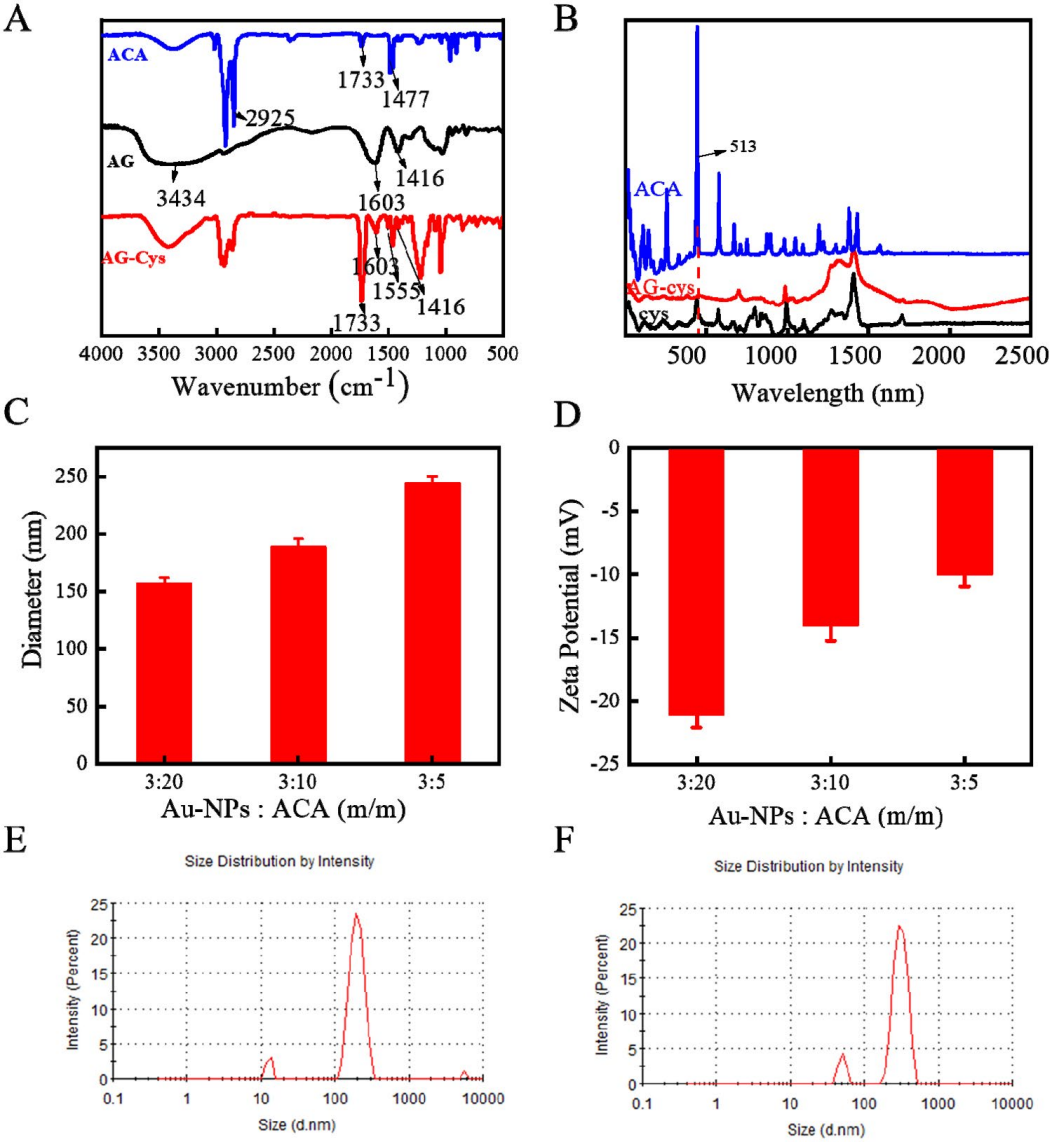
Structural characterization of ACA nanogels. (A) FTIR of ACA, AG, and AG-Cys; (B) Raman spectra of ACA, AG-Cys, and Cys; (C) size change of nanogels after loading AuNPs; and (D) zeta potential change of nanogels after loading AuNPs. Particle size distribution of ACA (E) and DOX@ACA (F).

Spherical ACA nanogels with a particle size of 150–200 nm and AuNPs with controlled particle size were prepared. Alginate nanogels (AG) were prepared with cysteine crosslinking using the double emulsion method (Sutthapitaksakul & Sriamornsak, 2019). AG showed the characteristic Fourier transform infrared (FTIR) spectra (Figure 1(A)) of a polysaccharide structure with broad peaks near 3434 cm^−1^ (hydroxyl stretching vibration of polysaccharides) and large absorption bands at 1603 and 1416 cm^−1^ (asymmetry) (Xu et al., 2020). The peak intensities at 1603 and 1416 cm^−1^ decreased significantly after the reaction, and the peaks shifted to 1637 cm^−1^ (C = O stretching (amide I)) and 1555 cm^−1^ (N-H in-plane bending (amide II)), indicating the formation of amide bonds between AG and Cys. The new AG-Cys peak at 1734 cm^−1^ could be associated with the ester bond (C = O stretching) of the diazonium sodium surfactant (Kwon & Kim, 2016). The Raman spectra (Figure 1(B)) showed the stretching vibration of the S–S bond of ACA-Cys and AG-Cys nanogels near 512 cm^−1^, indicating the successful crosslinking of AG (Kolosovas-Machuca et al., 2019). The absorption peak intensity of ACA-Cys and AG-Cys nanogels was relatively weak compared with the Raman spectra of Cys monomers, probably owing to the low S–S content in the nanogels. Therefore, the nanogels were successfully prepared. By varying the formulation parameters, the particle size could be adjusted (Figure 1(C)). For example, the nanogel size increased from 157 ± 3 nm to 243 ± 3 nm upon increasing the AuNP concentration from 15% to 60%.

### 3.2. Microstructure characterization of nanoparticles

**Figure 2.**
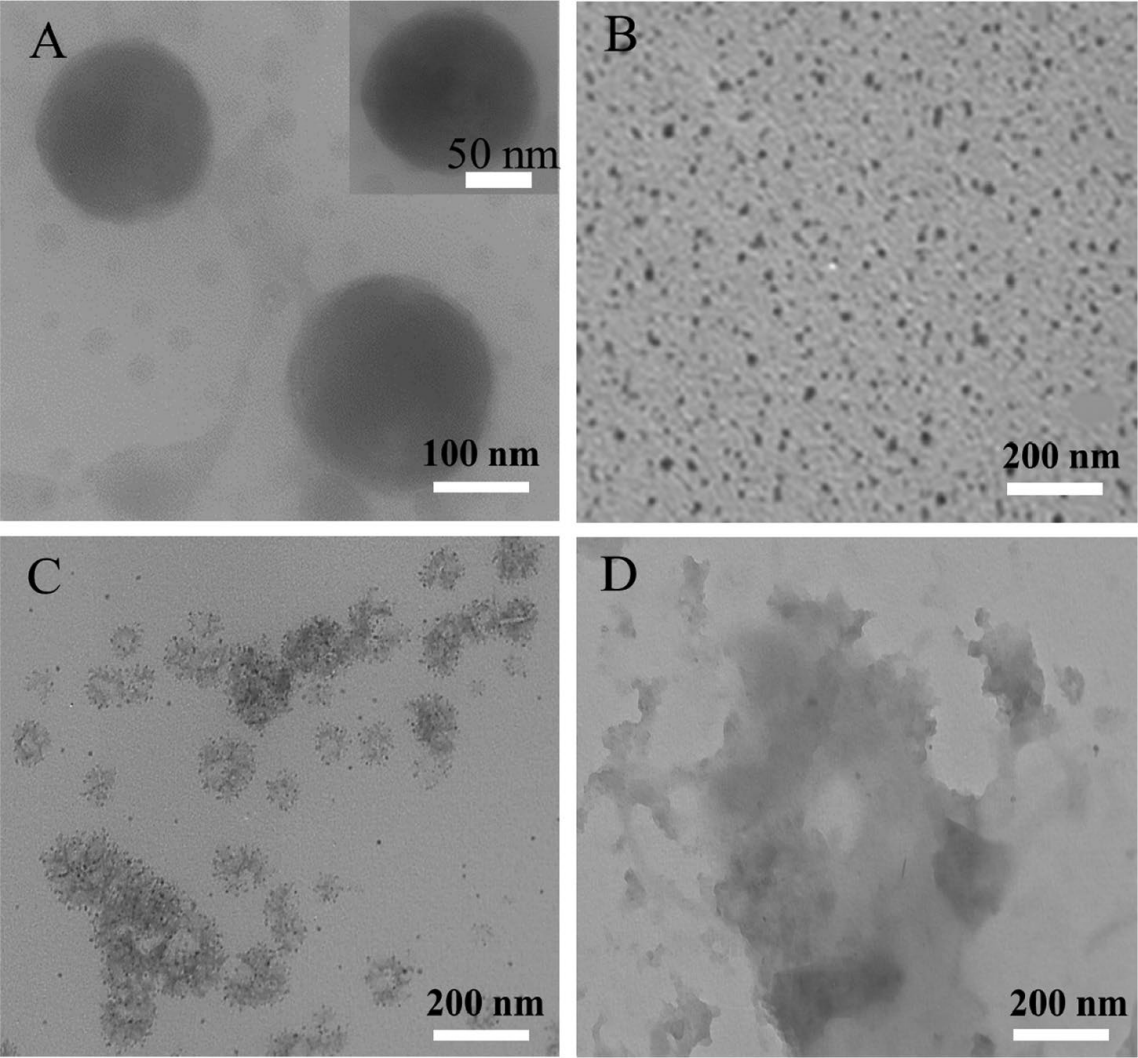
Microstructural characterization of ACA nanogels. TEM images of (A) pure AG nanogels, (B) AuNPs, (C) ACA nanogels, and (D) ACA nanogels degraded by alginate lyase.

To verify the binding of AuNPs and sodium alginate, we used TEM to characterize the microstructures of the AG, AuNP, and ACA nanogels. As shown in Figure 2(C), the size of the alginate nanogels was 200 ± 50 nm. The particle size of ACA nanogels increases from 150 to 250 nm with increasing amounts of AuNPs. The AuNPs were successfully prepared by the seeding growth method, and TEM indicated that the particle size was approximately 20 nm (Figure 2(B)). AuNPs were grown by electrostatic adsorption in situ on alginate to develop ACA nanogels. The ACA nanogel size was 100–200 nm, as determined by TEM (Figure 2(C)); this was comparable to those of clinically used nanomedicines with good therapeutic efficacy. Sodium alginate lyase can degrade sodium alginate, destroy the ACA nanogel framework, and release drugs. TEM images showed that after degradation, the sodium alginate structure was destroyed, and the spherical structure was lost (Figure 2(D)).

### 3.3. Drug loading and release properties of ACA nanogels

**Figure 3.**
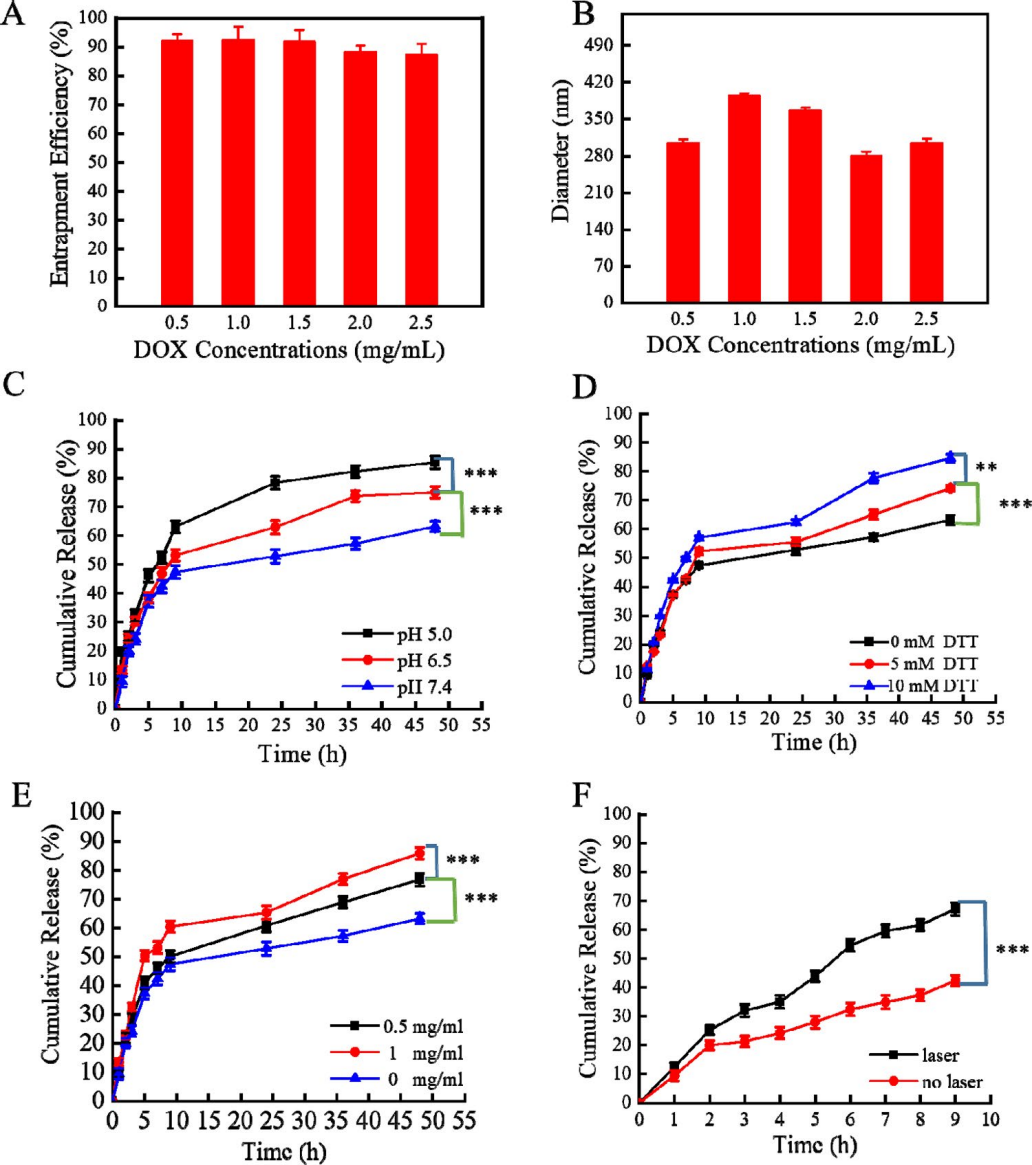
Drug loading and release properties of ACA nanogels: (A) entrapment efficiency of ACA nanogels; (B) particle size of ACA nanogels after DOX loading; (C) release behavior of DOX simulated *in vitro* at different pH values (5.0, 6.5, and 7.4); (D) DOX release at different DTT concentrations (0, 5, and 10 mM); (E) DOX release at different alginate lyase concentrations (0, 0.5, and 1 mg/mL); and (F) drug release ability of nanogels under laser irradiation (±standard deviation, *n* = 3, *p < .05, **p < .01, ***p < .001).

The negatively charged ACA nanogels were then used as a payload for DOX (Figure 3(A)). The loading rate of sodium alginate nanogels was higher than that of AuNP-loaded sodium alginate nanogels (Table 1). This was due to the positive charge of the AuNPs, which resulted in electrostatic repulsion toward the positively charged DOX. However, the ACA nanogels still showed a high encapsulation rate of ∼89.6% (Table 1). Therefore, our nanogels can serve as an excellent drug delivery platform.

**Table 1.** Hydrodynamic size, zeta potential, and drug-loading capacity of the nanogels.

Nanogels	Size (nm)	Zeta (mV)	EE (%)^a^	LC (%)^b^
AG/Cys	180.0 ± 17.1	−32.0 ± 9.2	–	–
DOX@AG/Cys	223.0 ± 12.0	−24.4 ± 13.6	91.3 ± 3.7	2.8 ± 0.9
ACA	214.0 ± 8.5	−19.3 ± 7.9	–	–
DOX@ACA	294.1 ± 11.4	−8.1 ± 5.0	89.6 ± 6.0	3.7 ± 0.6

a Encapsulation efficiency (*EE*) = *100* ×* W_t_*/*W_0_*, where *W_0_* is the weight of the loaded drug and *W_t_* is the weight of the packaged drug.

b Loading capacity (*LC*) = *100* ×* W_t_*/*W*, where *W_t_* is the weight of the loaded drug in the nanogel and *W* is the weight of the nanogel.

The controlled release of nanopharmaceuticals at a tumor site plays a vital role in antitumor therapy. Considering the complexity of human tissues, it is challenging to develop nanopharmaceutical platforms that can specifically identify tumors. Therefore, environmentally responsive drug release (e.g. drug release in response to different pH, redox, etc.) (Wang et al., 2019) has become the most promising controlled drug release approach. However, current drug release approaches mainly target the tumor microenvironment and have low accuracy. Drug release under external photothermal and enzyme stimulation could have higher accuracy.

The ACA nanogels synthesized in the current study facilitated tumor-targeting drug delivery in multiple ways. Based on the pH difference between the cancer tissue (pH 6.5) and the normal tissue (pH 7.4), DOX can be selectively released in the cancer tissue because ACA releases the drug under a low pH (Figure 3(C)); this ensures long-term anticancer activity (Knipe & Peppas, 2014). Furthermore, ACA nanogels selectively released DOX in response to redox. The glutathione (GSH) concentration in cancer tissues is higher than that in normal tissues, and cancer tissues are characterized by increased reducibility. Furthermore, the tumor intracellular microenvironment is acidic (pH 4.0–6.0), and its GSH concentration (1.0–10.0 mM) is 1000 times higher than that of the normal extracellular microenvironment (Raemdonck et al., 2009; Mohtaram et al., 2013; Zhang et al., 2020; Gong et al., 2021). The redox potential of the nanogels was evaluated by simulating a high-GSH tumor environment with DTT solution. The drug release efficiency increased with increasing DTT concentration (Figure 3(D)). Therefore, accurate drug release from the nanocarrier can be achieved in the high-GSH tumor environment.

Furthermore, the ACA nanogels are sensitive to sodium alginate lyase. For example, the drug release rate was significantly higher in the presence of 0.5 mg/mL lyase. Furthermore, DOX was almost completely released in 48 h in the presence of 1 mg/mL lyase (Figure 3(E)). After NIR laser irradiation (532 nm, 0.16 W/cm^2^), the drug release rate of ACA nanogels increased, and the release efficiency increased from 42.4 ± 1.8% to 67.2 ± 2.2% after 8 h (Figure 3(F)), indicating that the ACA nanogels have a photothermal effect along with drug delivery. The pH, redox, photothermal, and enzyme responses of the nanogels are promising for achieving multifactorial selective drug release in cancer tissues, thereby improving the antitumor effect and enhancing drug penetration.

### 3.4. Anticancer cytotoxicity of ACA nanogels in vitro

**Figure 4.**
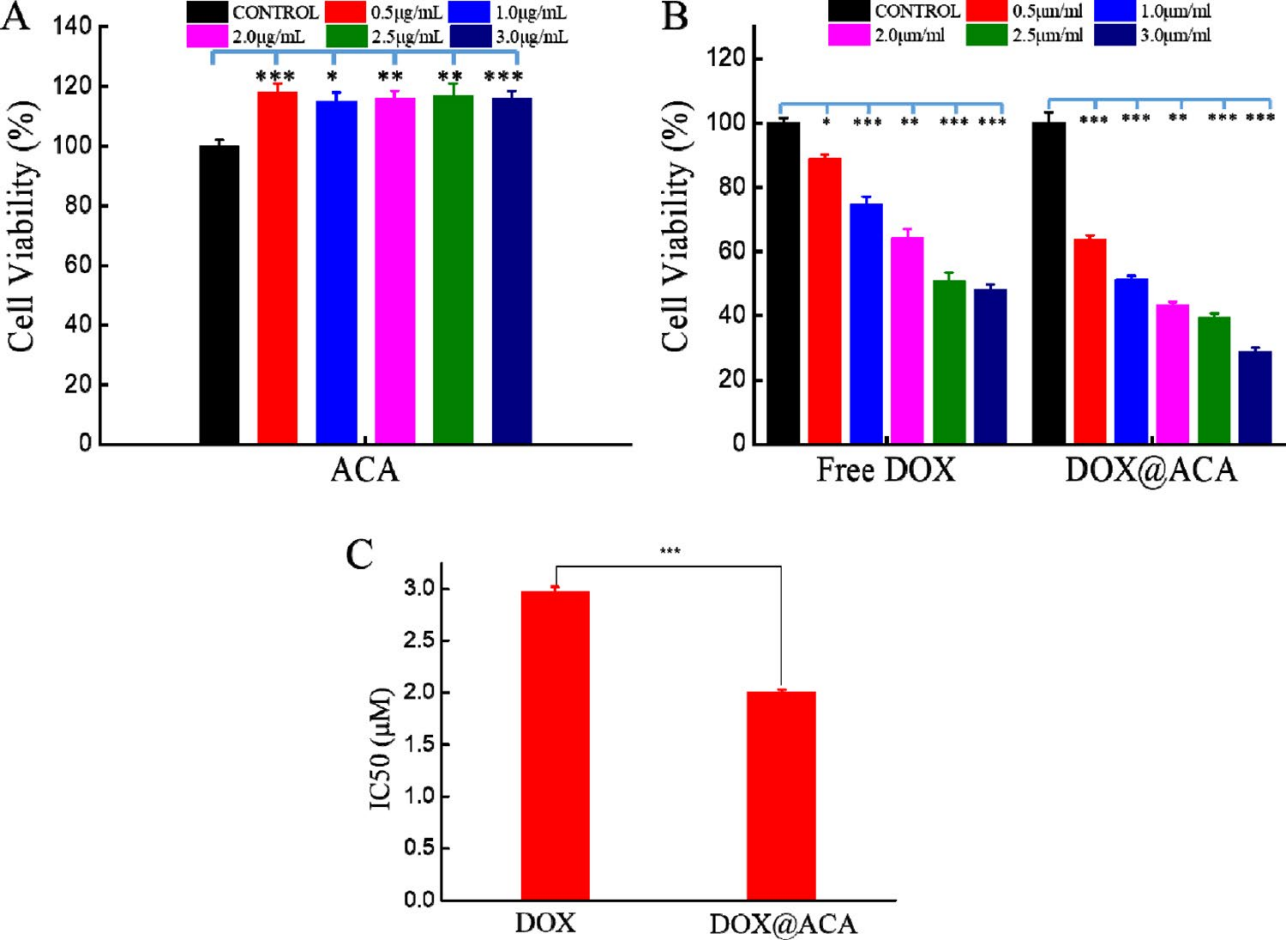
*In vitro* cytocompatibility and cytotoxicity: (A) ACA-treated A549 cell activity and (B) cell viability of A549 cells treated with free DOX and DOX@ACA (same DOX concentration) for 48 h. (C) IC50 values for ACA and DOX@ACA. (± standard deviation, *n* = 3, *p < .05, **p < .01, ***p < .001).

To verify the antitumor efficacy of the nanogels, we evaluated the cytotoxicity of ACA nanogels using A549 cells. A549 cells were incubated with ACA, DOX, and DOX@ACA at different concentrations for 48 h. ACA showed good biocompatibility (Figure 4(A)) and had no inhibitory effect on A549 cells. DOX@ACA was significantly more toxic to cancer cells than DOX (Figure 4(B)). DOX@ACA is highly toxic to cancer cells, indicating that DOX accumulates in these cells, possibly owing to the high drug load and controlled release of the nanogels. Free DOX was less toxic to A549 cells, possibly owing to multidrug resistance (MDR) in these cells. Therefore, ACA nanogels can deliver DOX with a high dose and increased therapeutic efficacy.

### 3.5. Antitumor effects in vivo

**Figure 5.**
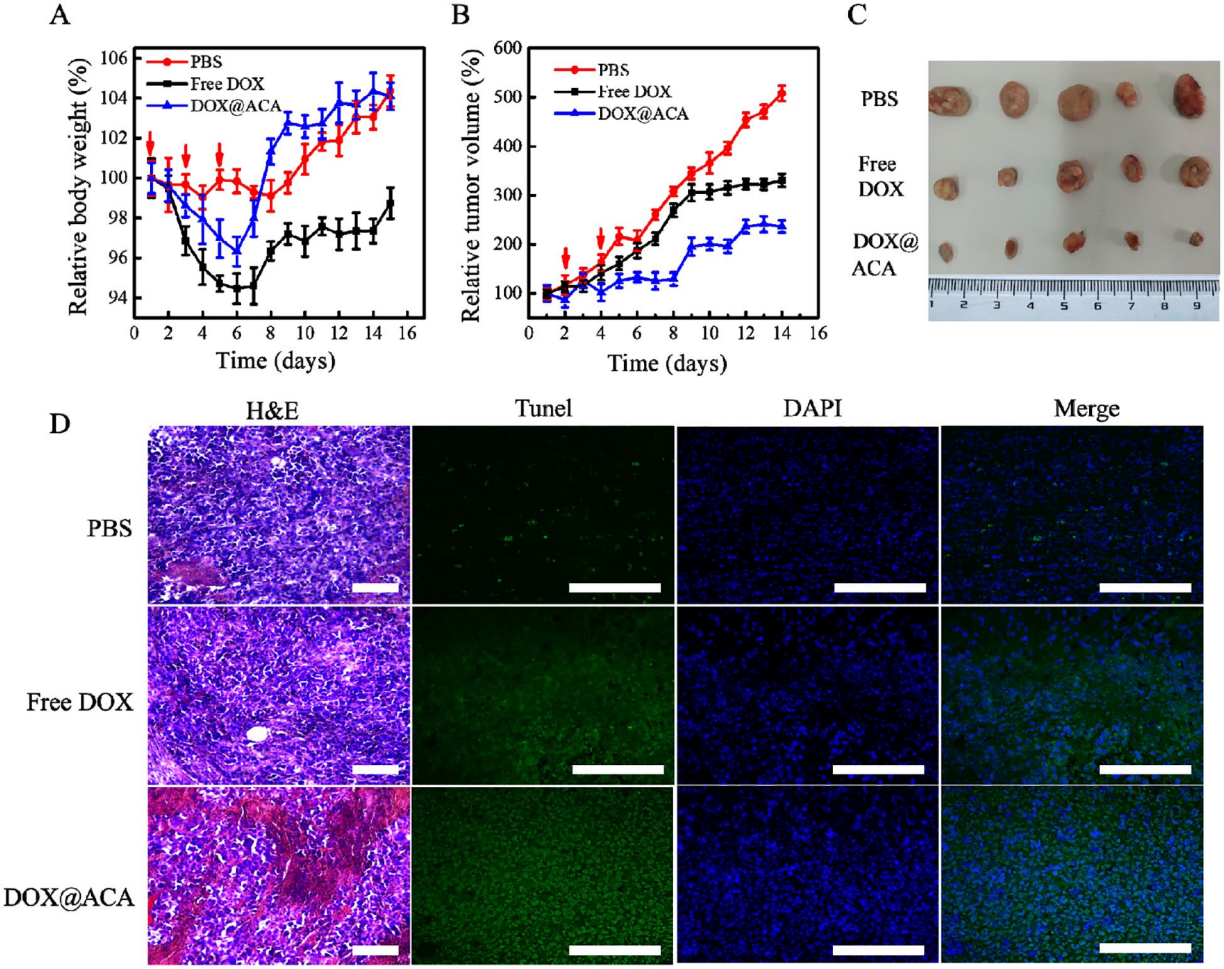
Inhibitory effect of DOX@ACA on tumors in mice: (A) weight changes in mice after treatment; (B) changes in tumor volume in mice after treatment; (C) representative images of tumor resection 15 days after PBS, DOX@ACA, and free DOX treatments; and (D) TUNEL staining of tumor sections: PBS, free DOX, and DOX@ACA treatments (scale: 200 μm).

Because DOX@ACA nanogels have a strong inhibitory effect on cancer cell proliferation, we further investigated the antitumor potential of the nanogels in vivo. To this end, 15 male C57BL/6J H22 mice with hepatocellular carcinoma were divided into three groups (irregular) and treated with free DOX, normal saline, and DOX@ACA. Free DOX and DOX@ACA were administered at 4 mg/kg. The tumor volume continued to increase in the mice treated with normal saline. Free DOX has a nontargeted effect on cancer cells and therefore has a low inhibitory effect on tumor growth (Figure 5(B)). DOX@ACA treatment had the strongest inhibitory effect on tumor growth owing to the drug release by nanogels in response to pH, redox, or enzymes in the tumor microenvironment and enhanced drug delivery in tumor cells. Notably, mice treated with free DOX lost weight rapidly, indicating the toxicity of free DOX to normal organs. However, mice treated with DOX@ACA gained weight and had a curve similar to that of the mice given normal saline (Figure 5(A)); this indicated the better biocompatibility and low biotoxicity of the nanogels. Histological observation of the tumor sections of the DOX@ACA nanogel treatment group revealed obvious damage, thus indicating increased cell apoptosis compared with the other groups (Figure 5(D)). To determine whether the cancer cells died through apoptosis, TUNEL staining was used to characterize the apoptosis of the cancer cells (Figure 5(D)). PBS-treated cancer tissues showed less apoptosis, and cancer cells treated with free DOX clearly showed apoptosis. The DOX@ACA treatment group showed more apoptosis than the free DOX and PBS treatment groups because DOX@ACA offered multiple responses and controlled drug release.

**Figure 6.**
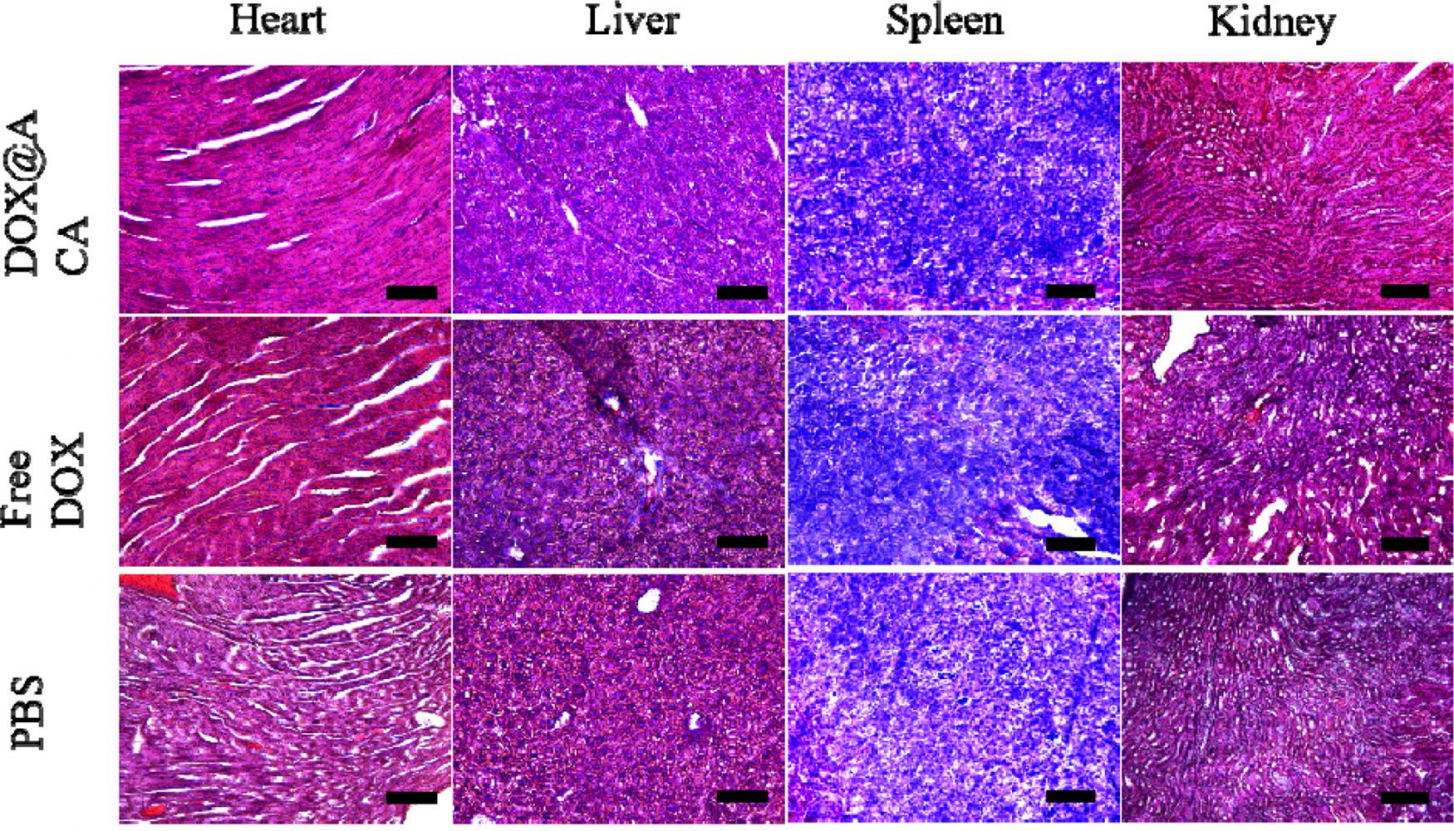
H&E-stained sections of vital organs (heart, liver, spleen, and kidney) (scale bar: 200 μm).

DOX treatment often causes additional damage to the organs. In this light, we examined tissues after DOX@ACA treatment with H&E staining. Neutrophils could be seen in the heart, liver, spleen, and kidney sections in the free DOX and blank control groups. In particular, tissues were significantly damaged in the free DOX group (Figure 6). On the other hand, the toxicity of DOX@ACA to various organs was significantly inhibited compared to that of free DOX. These findings indicated that DOX@ACA could kill a substantial proportion of cancer cells while protecting normal organs and tissues, thus suggesting that the drug toxicity and side effects could be efficiently reduced. This claim is also supported by the results of in vitro cell experiments and drug release experiments.

## 4. Conclusion

Nanogels improve drug delivery to tumor cells and therefore find broad application prospects in antitumor therapy. However, they cannot easily penetrate the extracellular matrix of the tumor owing to their large particle size, and accurate drug release remains difficult. Therefore, their clinical applications remain limited. Herein, we develop novel multistage drug delivery nanogels that afford improved tumor permeability and controlled delivery of anticancer agents in response to the tumor microenvironment. For this purpose, ∼100 nm multistage delivery nanogels with pH, redox, NIR stimulation, and enzyme responsiveness were prepared through the in situ growth of 20 nm AuNPs via an emulsion-aided crosslinking technique using a disulfide crosslinker. A negatively charged nanocarrier (ACA) is loaded with DOX through electrostatic interactions to produce a biodegradable nanocarrier (DOX@ACA). DOX@ACA has lower toxicity than DOX while affording slow drug release. In tumor tissues, the high pH and reductase microenvironment combined with the in vitro delivery of alginate and NIR irradiation drive drug release. The drug-containing 20 nm DOX combined with AuNPs with excellent photothermal effects effectively inhibited cancer cells. In vivo evaluations showed that multistage drug delivery using this nanogel effectively enhanced the antitumor activity and reduced the systemic toxicity of free DOX.

In summary, we have developed a multiresponse nanoplatform by crosslinking sodium alginate to cysteine and loading AuNPs in the resultant nanogels. It had a high drug loading capacity and achieved a strong anticancer effect through the combination of endogenous and exogenous stimuli for drug release. Sodium alginate with pH and enzyme response was crosslinked with cysteine to additional afford a redox response. DOX-AuNPs with good biocompatibility and an excellent photothermal effect were loaded on the nanogels. This nanoplatform facilitated drug release in the tumor microenvironment with low pH, redox, and high GSH; moreover, drug release and light-to-heat conversion could be realized through external photothermal stimulation and enzymes. The multiresponse and dual-stimulation release characteristics of this platform enabled the accurate and controlled release of anticancer drugs in the tumor microenvironment, thus achieving improved therapeutic efficacy and minimizing the side effects of free drugs. Therefore, the current study provides a potential antitumor therapy.
